# MicroRNA-429 inhibits neuroblastoma cell proliferation, migration and invasion via the NF-κB pathway

**DOI:** 10.1186/s11658-020-0202-9

**Published:** 2020-02-13

**Authors:** Xianjun Zhou, Hongting Lu, Fujiang Li, Xiwei Hao, Lulu Han, Qian Dong, Xin Chen

**Affiliations:** 1 Department of Pediatric Surgery, the Affiliated Hospital of Qingdao University, No.16 Jiangsu Road, Qingdao, 266000 Shandong China; 2 Department of Operation Room, the Affiliated Hospital of Qingdao University, No.59 Haier Road, Qingdao, 266000 Shandong China

**Keywords:** Neuroblastoma, NF-κB signaling, miR-429, IKKβ

## Abstract

**Background:**

MicroRNAs (miRNAs or miRs) can participate in the development and progression of neuroblastoma. Many studies have indicated that miR-429 can participate in tumor development. However, the mechanism underlying miR-429-mediated progression of neuroblastoma remains largely unclear.

**Methods:**

Colony formation and apoptosis assays were used to determine the effect of miR-429 on cell proliferation. Its impact on cell migration was determined using the wound-healing and Transwell assays. The target gene of miR-429 was confirmed via western blotting and luciferase reporter assays. A nude mouse xenograft model with miR-429 overexpression was used to assess the effect on tumor growth.

**Results:**

Our findings indicate that miR-429 is downregulated in neuroblastoma cell lines. We also found that it can induce apoptosis and inhibit proliferation in cells of those lines. MiR-429 can bind to the 3′-UTR of IKKβ mRNA and overexpression of IKKβ can reverse cell proliferation, blocking the effect of miR-429. Furthermore, miR-429 overexpression inhibited neuroblastoma growth in our nude mouse xenograft model.

**Conclusion:**

We provide important insight into miR-429 as a tumor suppressor through interaction with IKKβ, which is a catalytic subunit of the IKK complex that activates NF-κB nuclear transport. Our results demonstrate that miR-429 may be a new target for the treatment of neuroblastoma.

## Introduction

Neuroblastoma, which is a malignancy with high mortality, originates from neural crest pluripotent cells [1–3].. It has a high incidence in children under the age of 15 [4]. Treatment primarily involves surgery, but the recurrence rate is very high [5]. Although great progress has been made in clinical treatment, the survival rate of patients with metastatic neuroblastoma has not been improved [6]. Studies have shown that environmental endocrine disruptors may be involved in the disease’s progression [7]. Clarifying the mechanisms underlying neuroblastoma progression is necessary for development of more effective treatments.

NF-κB is upregulated in many blood and solid tumors [8], including neuroblastoma [9]. The NF-kB dimer, which acts as a transcription factor in the nucleus, is isolated in the cytoplasm in an inactive form that binds to an inhibitor of kappa B protein (IkB), usually IkBa. IKK phosphorylates IkBa, which is then degraded by the proteasome, allowing the NF-κB dimer to translocate [10]. IKBKB (IKKβ), which is a catalytic subunit of the IKK complex, activates NF-κB nuclear transport [11–13]. It is reported that activation of NF-κB signaling promotes tumorigenesis, progression and therapeutic resistance [14–18]. NF-kB can inhibit apoptosis of tumor cells by activating the transcription of anti-apoptotic genes [19].

Recent improvements in high-throughput gene expression analysis have revealed that microRNAs (miRNAs or miRs) can manipulate local or global gene expression via mRNA cleavage [20]. Endogenous miRNAs are involved in cell development, proliferation and apoptosis [21]. The occurrence of various tumors, including neuroblastoma, is often accompanied by dysregulated expression of specific miRNAs [22–24]. Previous studies have shown that miR-429 inhibits tumor development by binding to c-myc and PLGG1 in gastric and breast cancers [25, 26]. miR-429 also plays a tumor suppressing role in osteosarcoma [27]. However, few studies have investigated the detailed mechanisms of miR-429 in neuroblastoma.

In this study, we investigate the role of miR-429 in neuroblastoma, including its biological function in cells of the SK-N-SH and SH-SY5Y lines. Our findings show that miR-429 overexpression inhibits neuroblastoma cell proliferation and migration and promotes apoptosis. MiR-429 can directly target the 3′-untranslated region (3′-UTR) and suppress IKKβ in vivo and in vitro. Thus, miR-429 might play an important role in inhibiting the progression of neuroblastoma.

## Materials and methods

### Cell culture and transfection

Human neuron cells (ScienCell, cat. no. 1520) were cultured in Dulbecco’s modified Eagle’s neuronal medium (DMENM; cat. no. 1521). Cells of the human neuroblastoma cancer lines IMR-32, SK-N-SH and SH-SY5Y were cultured in Dulbecco’s modified Eagle’s medium (DMEM; Thermo Fisher Scientific, cat. no. 11995040) supplemented with 10% fetal bovine serum at 37 °C in a humidified incubator with 5% CO_2_. MiR-429 mimics, control, inhibitor and plasmids were purchased from GenePharma. Cells were transfected with the miR-429 mimic, inhibitor or pcDNA3.1-IKKβ using Lipofectamine 3000 (Thermo Fisher Scientific) according to the manufacturer’s instructions.

### Extraction of total RNA and quantitative real-time PCR

Total RNA was extracted with Trizol (Invitrogen). cDNA was synthesized using M-MLV reverse transcriptase (Promega), and mRNA quantitative detection was performed using a StepOne Real-Time PCR system and fast SYBR Green Master Mix (Applied Biosystems). Primers were synthesized by Invitrogen. The PCR conditions were: 94 °C for 2 min, followed by 30 cycles of 94 °C for 30 s, 60 °C for 30 s and 72 °C for 1 min, and finally 72 °C for 10 min.

The relative expression level of mRNA was calculated using the 2^-ΔΔCq^ method. The primers were: miR-429 forward, 5′-CTAACCGACCCAGAAATAAGCG-3′ and reverse, 5′-TATCGGCCATGCTCCGGAAAGG-3′; U6 forward, 5′-GATTACAGC CGAACGTGTAGGAA-3′ and reverse, 5′-AGCTTGATCGTTTCTCTGGCCACC-3′; IKKβ forward, 5′-GCCAGAAAACATCGTCCT-3′ and reverse, 5′-CACCGTTCCA TTCAAGTC-3′; cyclinD1 forward, 5′-AGGAGAACAAACAGATCA-3′ and reverse, 5′-TAGGACAGGAAGTTGTTG-3′; IL-8 forward, 5′-AACATGACTTCCA AGCTGGCCG-3′ and reverse, 5′-CAGTTTTCCTTGGGGTCCAGAC-3′; Bcl-2 forward, 5′-AGCAGCAAGTAGGTGTCCCAG-3′ and reverse, 5′-CTCCACGCCAT CTTGCTTCT-3′; and GAPDH forward, 5′-TCCAGAGTGCAAGGCTTCAG-3′ and reverse, 5′-GACAGCACGCAGTAGCAGTAG-3′.

### Western-blotting

The protein in the cell lysates was separated via SDS-PAGE and transferred to nitrocellulose membranes (micropores). Primary antibodies (Abcam) targeting the following proteins were applied: IKKβ (cat. no. ab124957, 1:2000), cyclinD1 (cat. no. ab16663, 1:2000), Bcl-2 (cat. no. ab59348, 1:2000), IL-8 (cat. no. ab18672, 1:2000), and GAPDH (cat. no. ab9485, 1:1000). An IRDye-labeled donkey anti-mouse or rabbit anti-IgG (Licor Biosciences) was used as the secondary antibody, and the membrane was assayed with an Odyssey Infrared Imaging System (Gene Company Limited).

### Cytotoxicity assay

Cell proliferation was determined using a Cell Counting Kit-8 (CCK-8). Cells were seeded in 100 μl of medium supplemented with 10% FBS at 5 × 10^4^ cells/well in 96-well plates. After 48 h incubation,10 μl of CCK-8 reagent was added to each well and the cells were cultured for 1 h at 37 °C in a humidified incubator with 5% CO_2_. The absorbance at 450 nm was measured with a microplate reader (Bio-Tech Company).

### Colony formation assay

After the cells were transfected with miR-429 mimic or inhibitor, they were cultured in a 6-well plate for 10 days. The colonies were fixed with methanol for 30 min and stained with 1.0% crystal violet for 20 min.

### Scratch-healing migration assay

Briefly, cells were seeded at 5 × 10^4^ cells/well in 24-well plates and cultured for 24 h. Wounds were created using a 10-μl pipette tip. Wound healing was assessed after 24 h. We randomly selected 5 locations for assessment and photographing. Images were obtained with a Zeiss Axiovert 200 microscope.

### Cell invasion assay

Briefly, 5 × 10^4^ cells were added into the upper chamber of a Transwell, and then, 0.7 ml DMEM was added to the lower chamber. Cells were cultured for 24 h at 37 °C in a humidified incubator with 5% CO_2_. After treatment, the cells were fixed with methanol for 30 min and stained with 1.0% crystal violet for 20 min. The number of invasive cells penetrating the Matrigel was recorded.

### Cell apoptosis assay

Cells transfected with the miR429 mimic, inhibitor or control were incubated for 48 h and then collected. Cells were analyzed for double staining with FITC Annexin V and PI using a FITC Annexin V Apoptosis Detection Kit and CellQuest software (both from BD Biosciences) according to the manufacturer’s protocol.

### Luciferase reporter assay

The 3′-UTR of IKKβ was synthesized and inserted into pMIR-REPORT. Cells were transfected with miR-429 mimic as indicated 24 h before transfection with pMIR-REPORT-IKKβ. Luciferase activity was measured with a Dual-Luciferase Reporter Assay System (Promega).

### Immunohistochemistry

Tumor sections were incubated with antibody against IKKβ (1:150) overnight, washed three times with PBS containing 0.05% Tween, incubated for 2 h at room temperature, and washed three times with PBS containing 0.05% Tween. The sections were then visualized with 3,3′-diaminobenzidine (DAB) substrate and counterstained with hematoxylin QS. Ten fields were selected for imaging under a microscope (Carl Zeiss).

### In vivo tumorigenicity assay

Lentiviruses carrying empty vector (NC) and miR-429 expression vector (miR-429 mimics) were used to infect SH-SY5Y cells that showed stable expression in this study. These were used in an in vivo tumorigenicity assay.

Briefly, 4- to 5-week-old BALB/c nude mice were purchased from Beijing HFK Biotechnology. The mice were housed in a pathogen-free animal facility and randomly assigned to the control or experimental group (five mice per group). Then, 2 × 10^6^ of the NC or miR-429 mimic SH-SY5Y cells were resuspended in 200 μl of PBS and subcutaneously injected into the nude mice. The tumor diameter and size were measured every 3–4 days to monitor tumor formation. After euthanasia, the tumor was recovered and the wet weight of each tumor was examined.

### Statistical analysis

All statistical analyses were performed using SPSS 17.0 (SPSS, Chicago, USA) using either one-sample *t-*test or one-way ANOVA. All data are presented as the mean ± S.E.M. A *p* value of less than 0.05 was indicated with *, and a p value of less than 0.01 was indicated with **.

## Results

### miR-429 was underexpressed in neuroblastoma cells

**Fig. 1:**
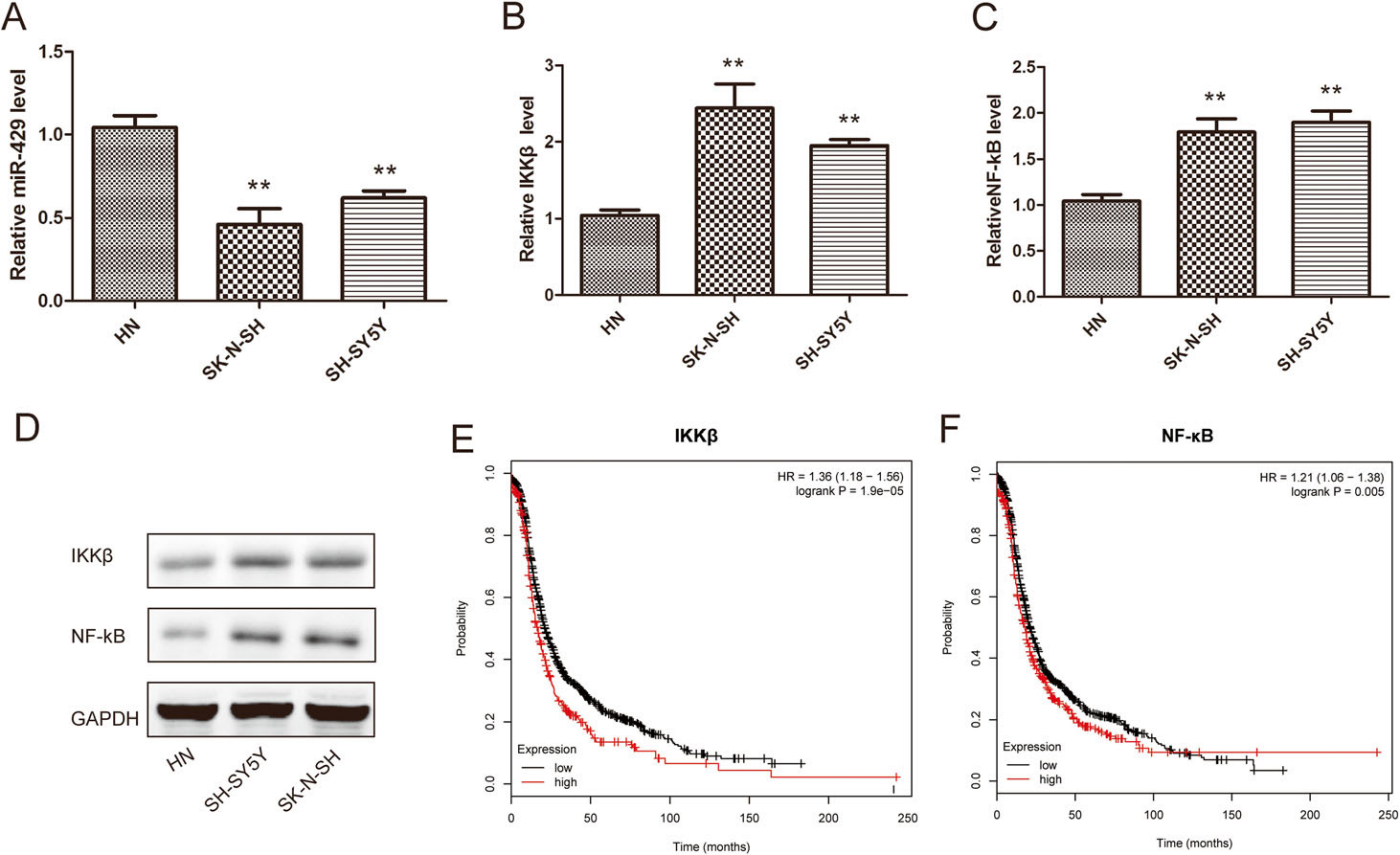
miR-429 was underexpressed in NB cells*.***a** – Quantitative RT-PCR was performed to determine the expression of miR-429 in neuroblastoma cells (SH-SY5Y, SK-N-SH) and human neurons (HNs). **b** through **d** – The expression levels of IKKβ and NF-κB in NB cells (SH-SY5Y, SK-N-SH) and HNs. **e** and **f** – Kaplan–Meier analysis of the overall survival of NB patients in the TCGA database with high versus low IKKβ and NF-κB expression. U6 and GAPDH were used as the loading controls. Error bars represent the means ± SEM of at least three independent experiments. n.s.: not significant; *p < 0.05 and **p < 0.01 vs. the control group

We first compared miR-429 expression in neuroblastoma cell lines and human neuronal cells. Our results showed that miR-429 expression was significantly lower in neuroblastoma cell lines than in normal cells (Fig. 1a).

Interestingly, IKKβ and NF-κB also showed differential expression between neuroblastoma cell lines and human neurons. The expressions of both were significantly higher in neuroblastoma cell lines (Fig. 1b through d). Furthermore, neuroblastoma patients with higher NF-κB and IKKβ expressions had a poorer prognosis than those with lower NF-κB and IKKβ expressions (Fig. 1e and f). These results suggest that miR-429 and IKKβ play an important role in the development of neuroblastoma.

### miR-429 inhibition accelerates the proliferation, migration and invasion of neuroblastoma cells in vitro

**Fig. 2:**
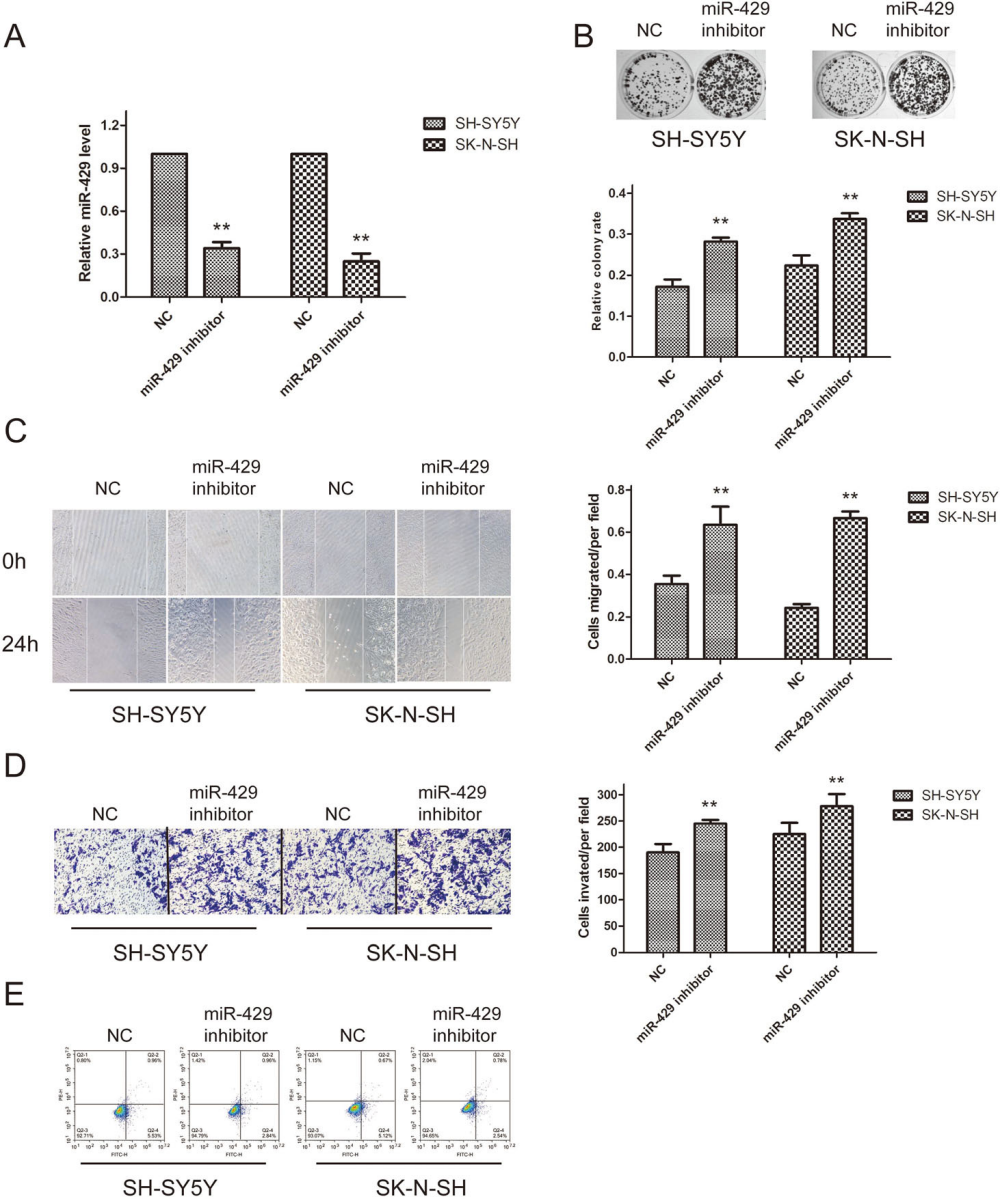
miR-429 inhibition accelerates the proliferation, migration and invasion of neuroblastoma cells in vitro. **a** – Downregulation of miR-429 by transfection with miR-429 inhibitor in SH-SY5Y and SK-N-SH cells. **b** – Colony formation assays were applied to determine the effect of miR-429 knockdown on SH-SY5Y and SK-N-SH cell proliferation ability. **c** and **d**– Wound-healing assays and Transwell assays were employed to examine the effect of miR-429 knockdown on the migration capacity of SH-SY5Y and SK-N-SH cells. **e** – The percentage of apoptotic cells was determined by flow cytometry. Error bars represent the means ± SEM of at least three independent experiments. n.s.: not significant; *p < 0.05 and **p < 0.01 vs. the control group

We then knocked down miR-429 in SK-SY5Y and SK-N-SH cells. The expression of miR-429 was significantly downregulated at the RNA level (Fig. 2a). Colony formation assays showed that miR-429 inhibition significantly increased the rate of cell proliferation (Fig. 2b). Scratch and invasion assay results showed that the cell migration capability significantly increased after transfection with the miR-429 inhibitor (Fig. 2c). A Matrigel invasion assay also showed that the invasion capacities of SK-SY5Y and SK-N-SH cells significantly increased after transfection with the miR-429 inhibitor (Fig. 2d). The apoptosis assessment results demonstrated that miR-429 knockdown suppressed SK-SY5Y and SK-N-SH cell apoptosis (Fig. 2e). These results suggest that miR-429 can suppress the progression of neuroblastoma.

### miR-429 overexpression blocked the proliferation, migration and invasion of neuroblastoma cells in vitro

**Fig. 3:**
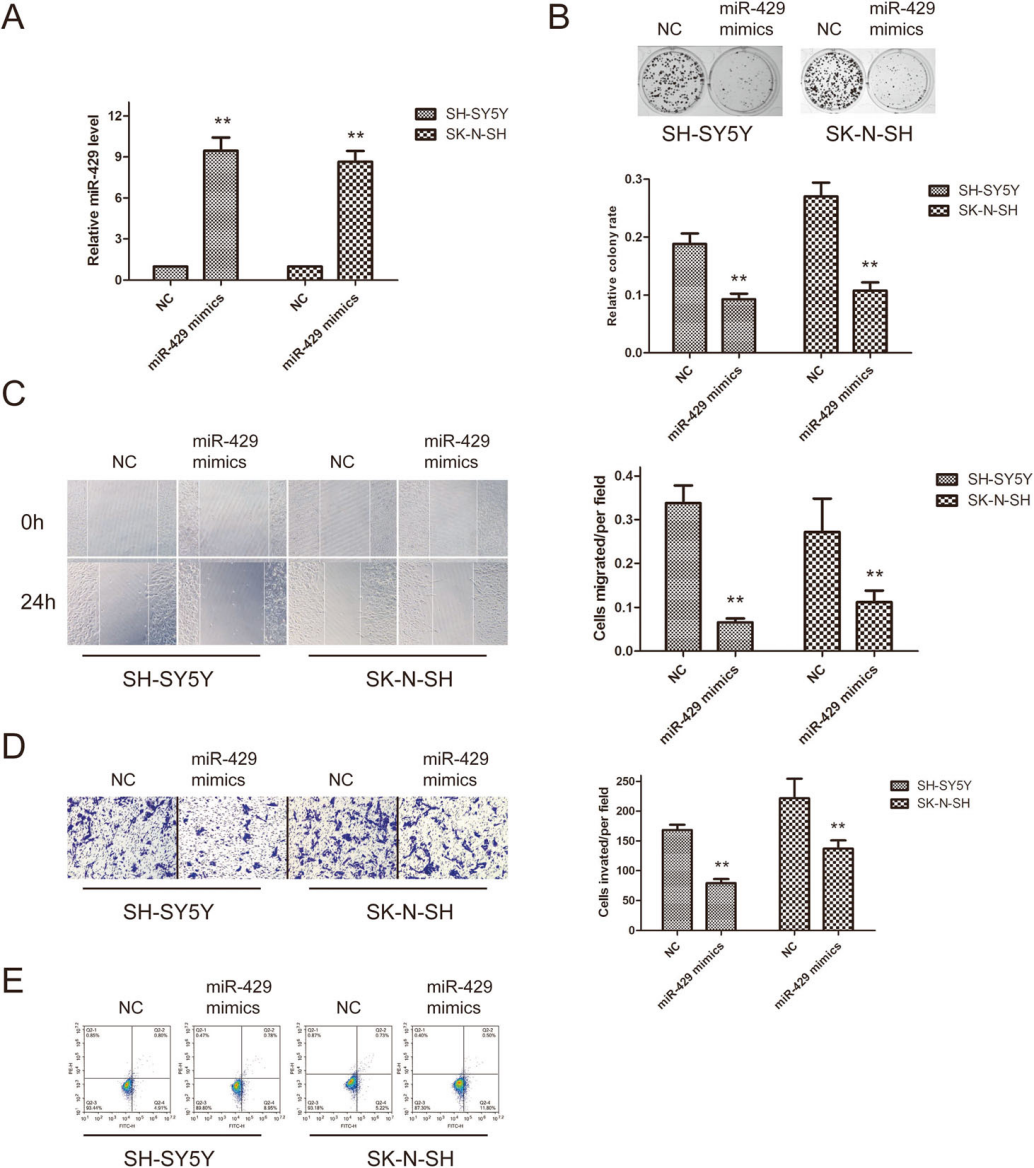
MiR-429 overexpression blocked the proliferation, migration and invasion of neuroblastoma cells in vitro. **a**– Overexpression of miR-429 in SH-SY5Y and SK-N-SH cells transfected with the miR-429 mimic. **b** – Colony formation assays were applied to determine the effect of miR-429 overexpression on SH-SY5Y and SK-N-SH cell proliferation ability. **c** and **d** – Wound-healing (**c**) and Transwell (**d**) assays were employed to examine the effect of miR-429 overexpression on the migration capacity of SH-SY5Y and SK-N-SH cells. **e** – The percentage of apoptotic cells was determined by flow cytometry. Error bars represent the means ± SEM of at least three independent experiments. n.s.: not significant; *p < 0.05 and **p < 0.01 vs. the control group

Next, SK-SY5Y and SK-N-SH cells were transfected with the miR-429 mimic. The miR-429 level was significantly higher in the transfected cells (Fig. 3a). Furthermore, colony formation assays showed that the cell proliferation rate was inhibited (Fig. 3b). Similarly, scratch and invasion assays indicated that miR-429 overexpression inhibited the migration and invasion abilities of SH-SY5Y and SK-N-SH cells (Fig. 3c and d). Flow cytometric analysis indicated that miR-429-transfected SH-SY5Y and SK-N-SH cells exhibited an enhanced apoptosis rate compared with control cells (Fig. 3e) These results further demonstrate that miR-429 is able to suppress neuroblastoma progression.

### IKKβ was identified as a target gene of miR-429

**Fig. 4:**
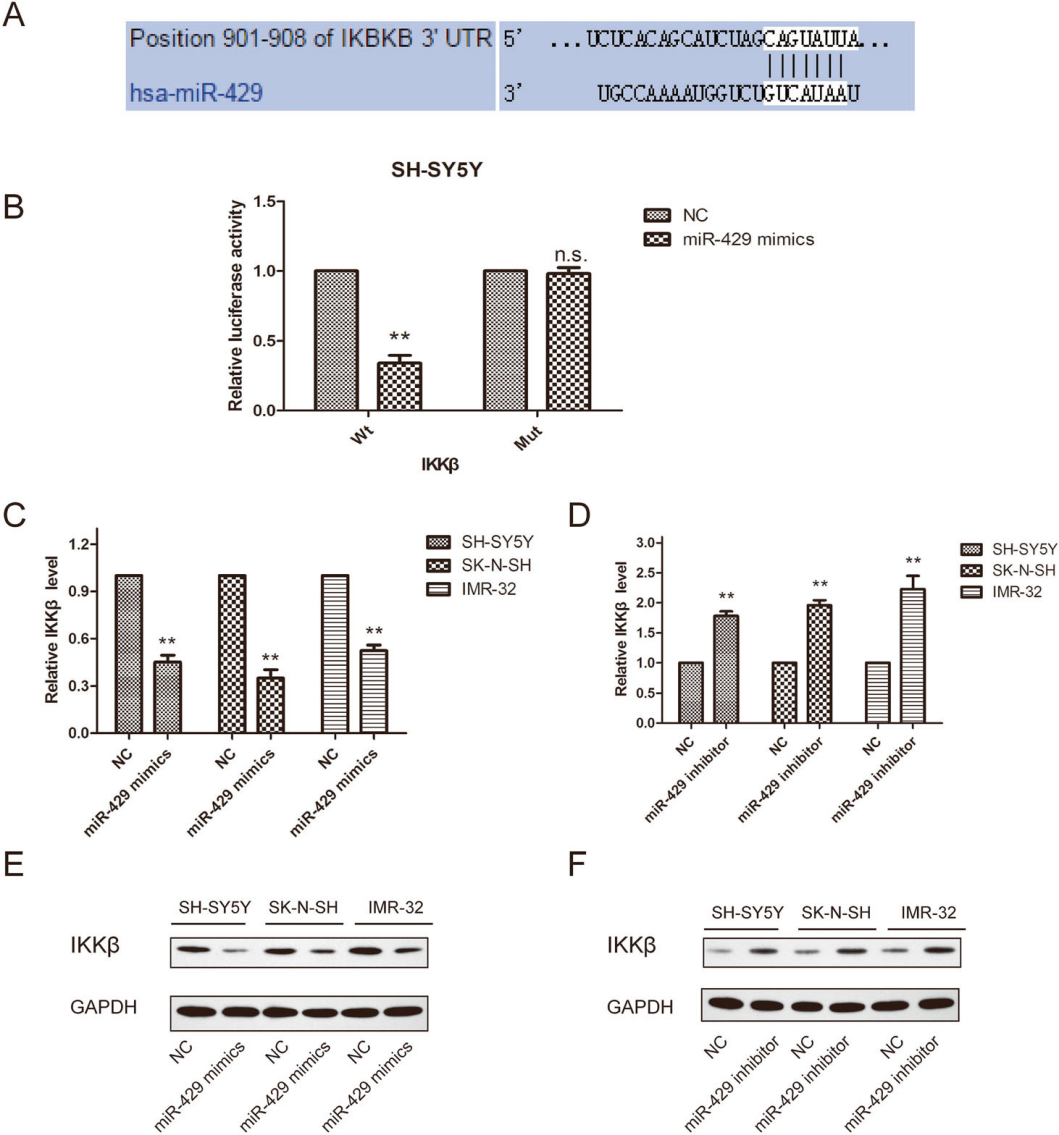
IKKβ was identified as a target gene of miR-429. **a** – The binding sites between miR-429 and IKKβ. **b** – The miR-429 mimic decreased the luciferase reporter activity of IKKβ. **c** – Downregulation of miR-429 expression increased the expression of IKKβ at the mRNA level in SH-SY5Y, SK-N-SH and IMR-32 cells. **d** – Overexpression of miR-429 inhibited the expression of IKKβ at the mRNA level in SH-SY5Y, SK-N-SH and IMR-32 cells. **e** – Western blot showing IKKβ protein expression levels in SH-SY5Y, SK-N-SH and IMR-32 cells after transfection with NC-mimic, miR-429 mimics. **f** – Western blot showing IKKβ protein expression levels in SH-SY5Y, SK-N-SH and IMR-32 cells after transfection with NC-inhibitor and miR-429 inhibitor. GAPDH was used as the endogenous control. Error bars represent the means ± SEM of at least three independent experiments. N.S.: not significant; *p < 0.05 and **p < 0.01 vs. the control group

Based on miR target analysis using the websites targetscan, PicTar and miRanda, miR-429 was found to be a potential regulator of IKKβ (Fig. 4a). Luciferase reporter assays showed that miR-429 overexpression can decrease the luciferase activity of the wild-type (Wt) 3′-UTR of IKKβ but did not affect luciferase activity of the mutant (Mut; Fig. 4b). Moreover, in SH-SY5Y, SK-N-SH and IMR-32 cells, the expression of IKKβ was regulated by miR-429 at the mRNA and protein level. Our results showed that the miR-429 mimic significantly inhibited the expression of IKKβ (Fig. 4c and e), whereas silencing of miR-429 significantly increased IKKβ expression (Fig. 4d and f). These data suggest that IKKβ is a target of miR-429 in neuroblastoma cells.

### miR-429 inhibited the NF-κB pathway

We investigated the molecular mechanisms that may be involved in the anticancer effects of miR-429. The NF-κB signaling pathway is often found to be abnormally activated in neuroblastoma, promoting cell proliferation and inhibiting apoptosis [25, 26]. We explored whether miR-429 regulates NF-κB activity.

**Fig. 5:**
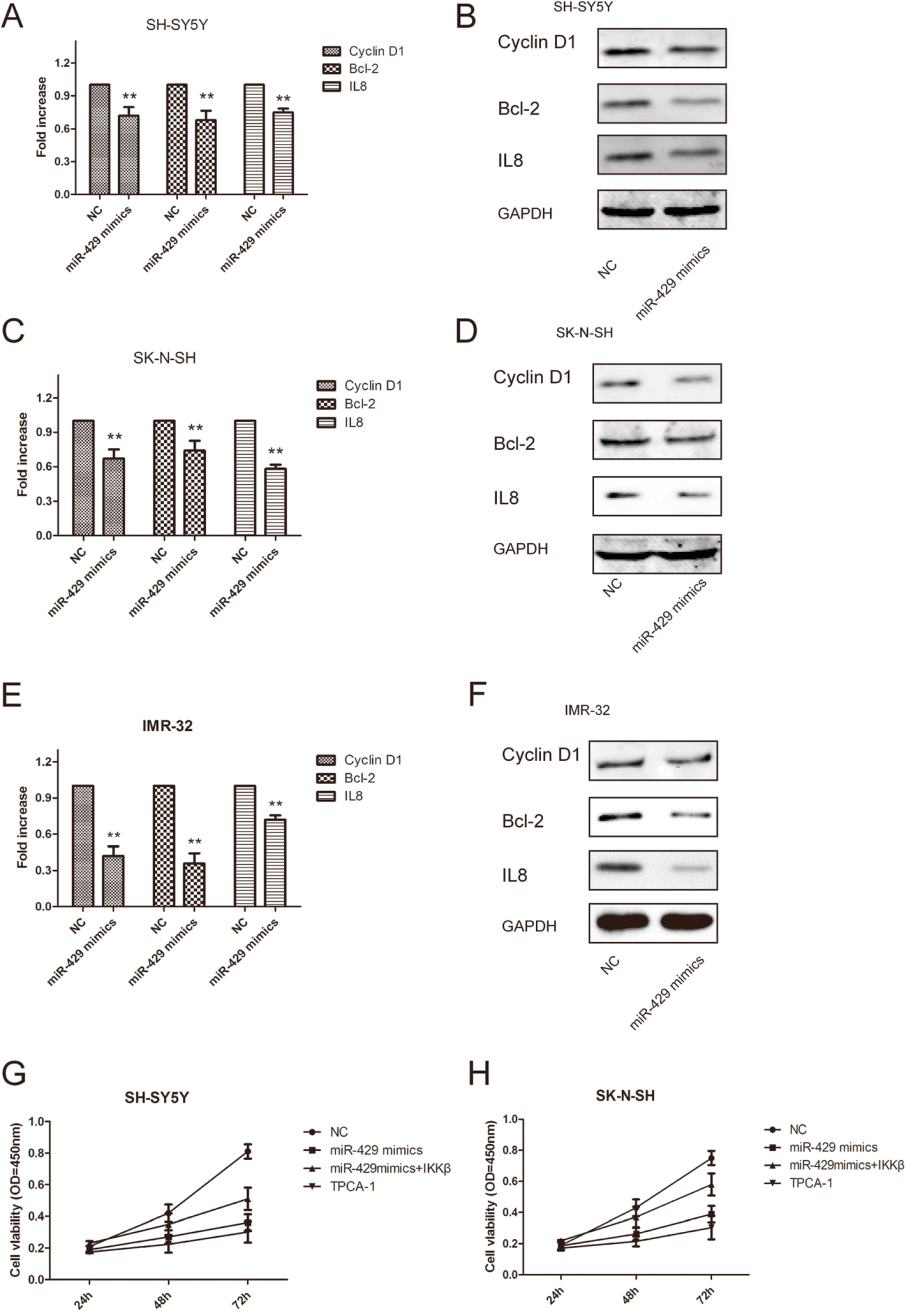
MiR-429 inhibited the NF-κB pathway. **a**, **c** and **e** – Relative mRNA expression of NF-κB-regulated genes in SH-SY5Y (**a**), SK-N-SH (**c**) and IMR-32 (**e**) cells. GAPDH was used as a loading control. **b**, **d** and **f** – Western blot showing NF-κB-regulated gene protein expression levels in SH-SY5Y (**b**), SK-N-SH (**d**) and IMR-32 (**f**) cells. **g** and **h** – Overexpression of IKKβ attenuated the anticancer effect of miR-429 in SH-SY5Y and SK-N-SH cells. Error bars represent the mean ± SEM of at least three independent experiments. N.S.: not significant; *p < 0.05 and **p < 0.01 vs. the control group

The mRNA expression levels of three NF-κB target genes were significantly lower in SH-SY5Y, SK-N-SH and IMR-32 cells transfected with the miR-429 mimic (Fig. 5a, c and e). Under the same conditions, the protein expression of the NF-κB target genes cyclin D1, Bcl-2 and IL8 was lower in SH-SY5Y, SK-N-SH and IMR-32 cells (Fig. 5b, d and f).

Further investigations were performed to study whether overexpression of IKKβ could attenuate the anticancer effect of miR-429. MTT assays showed that overexpression of IKKβ could significantly attenuate the anticancer effect of miR-429 in SH-SY5Y and SK-N-SH cells (Fig. 5g and h), suggesting that miR-429 inhibited the proliferation of neuroblastoma cells in part by targeting IKKβ.

We then treated neuroblastoma cells with the IKKB inhibitor TPCA-1 and obtained the same results as observed after transfection with the miR-429 mimic. Our results indicate that IKKβ plays an important role in miR-429-mediated NF-κB activation.

### miR-429 overexpression inhibits neuroblastoma growth in a nude mouse xenograft model

To address the role of miR-429 in neuroblastoma tumorigenesis in vivo, we established miR-429 SH-SY5Y stable cell lines (miR-429 mimics) to study their biological functions in a murine model. We initiated tumor growth by subcutaneously injecting 2 × 10^6^ miR-429 mimics cells into BALB/c mice and monitored tumor growth by measuring the dimensions.

**Fig. 6:**
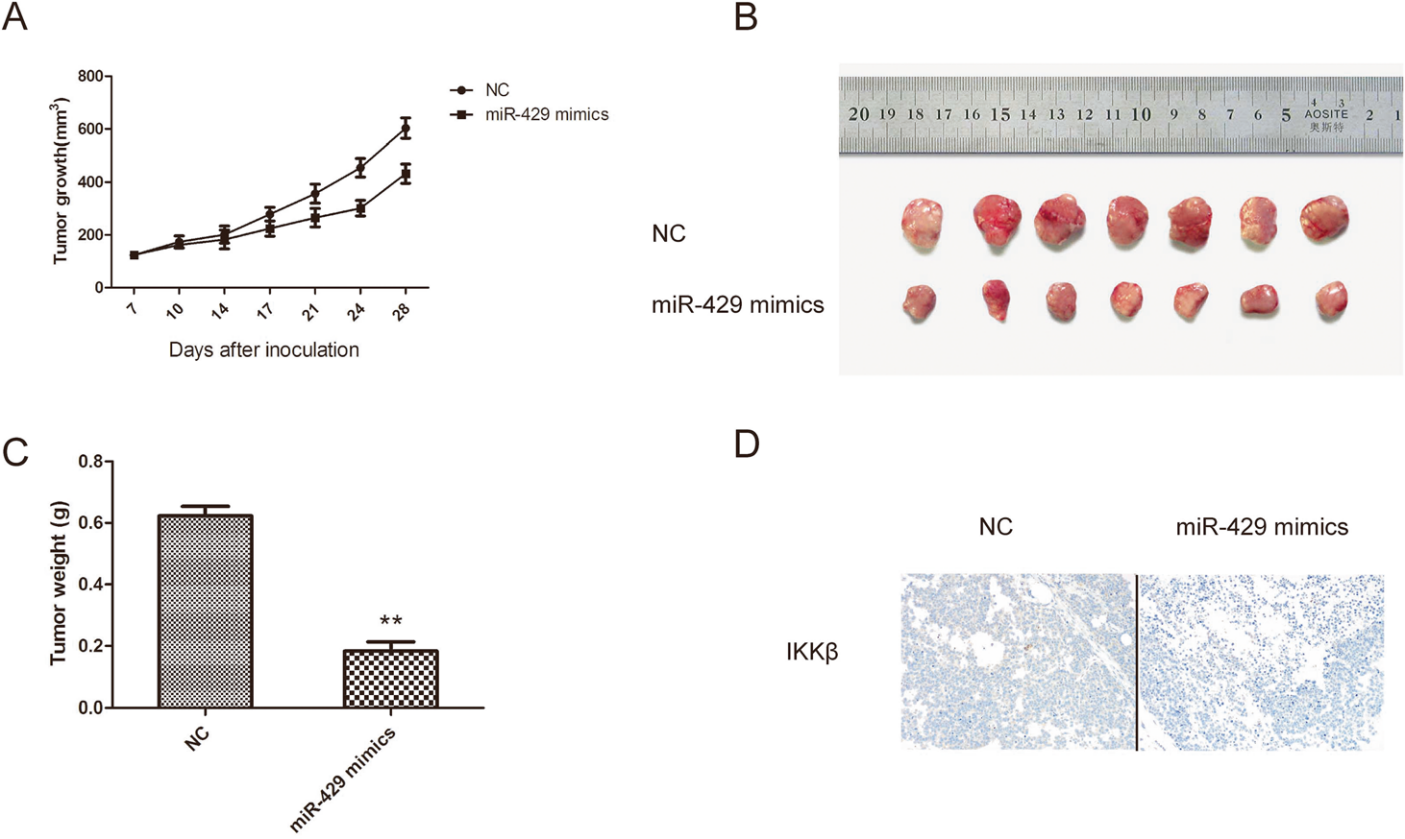
miR-429 overexpression inhibited neuroblastoma growth in a nude mouse xenograft model. The mice were randomly assigned to the control or experimental group (five mice per group). Then, 2 × 10^6^ of the NC or miR-429 mimic SH-SY5Y cells subcutaneously injected into the nude mice. **a** – Tumor volume was calculated twice weekly. **b** – Photographs of tumors derived from NC and miR-429 mimic cells in nude mice. **c** – Weights of tumors. **d** – IKKβ expression was examined in xenografts that were treated with or without miR-429 mimic-transfected cells. Error bars represent the means ± SEM of at least three independent experiments. N.S.: not significant; *p < 0.05 and **p < 0.01 vs. the control group

The tumors formed from SH-SY5Y cells transfected with the miR-429 mimic grew significantly more slowly than those from control cells and had lower weights (Fig. 6a through c).

In addition, immunohistochemical staining was performed to determine the expression of IKKβ in mouse tumor tissues. IKKβ expression decreased in the xenograft tumors after treatment with the miR-429 mimics (Fig. 6d). These data suggest that miR-429 plays an important role in the development of neuroblastoma.

## Discussion

Despite advances in treatment, there have been limited improvements in the survival of patients with neuroblastoma. This is mainly due to a lack of early detection methods. In recent years, studies of epigenetic biomarkers, such as miRNA expression, suggest that epigenetic changes may be linked to neuroblastoma. It has been reported that miRNAs are involved in a variety of cellular processes and diseases through direct binding to the 3′-UTR of target mRNAs. Understanding the role of miRNAs that are aberrantly expressed in neuroblastoma can help us to understand the underlying mechanisms and improve therapeutic approaches.

In this study, we evaluated the expression level of miR-429 in neuroblastoma cells and human neurons. Our results suggest that miR-429 may play an important role in cervical carcinogenesis. We showed that overexpression of miR-429 could obviously inhibit neuroblastoma cell metastasis ability and proliferation, while knockdown of miR-429 had the opposite effects. This shows that miR-429 might have critical roles in neuroblastoma cells.

MiR-429 was found to play an oncogenic role in various endometrial carcinomas. However, miR-429 was also found to have a tumor-suppressive role in some cancers. For example, miR-429 inhibited the proliferation and migration of gastric cancer cells [28–30]. These suppression results are consistent with our results in neuroblastoma.

The IKK complex directly activates NF-κB, which is regulated by miRNAs. The canonical IKK complex consists of three major subunits: IKKα, IKKβ, and IKKγ. A large number of studies have shown that IKKβ is associated with the occurrence of a variety of tumors. It has been reported that IKKβ is negatively regulated by miR-199a [31] and IKKα is negatively regulated by miR-16 [32] to reduce NF-κB activity.

In our study, we demonstrated that IKKβ promotes oncogenic activity and may mediate the effects of miR-429 on malignancy. We confirmed potential binding sites between miR-429 and IKKβ through bioinformatics analysis. Luciferase reporter assays indicated that miR-429 can bind to the 3′-UTR region of IKKβ. We then found that miR-429 overexpression could decrease the expression of IKKβ at the mRNA and protein level. MTT assays showed that overexpression of IKKβ could significantly attenuate the anticancer effect of miR-429. The mRNA and protein expressions of the three NF-κB target genes significantly decreased in SH-SY5Y and SK-N-SH cells transfected with the miR-429 mimic.

Based on this, we speculate that miR-429 may influence the activation of the IKKβ/NF-κB pathway. We revealed that during the activation of the NF-κB pathway, miR-429 reduced the level of activated NF-κB by suppressing IKKβ. It has been reported that many other signaling pathways are also involved in tumor migration and other processes, such as the Wnt signaling pathway [33, 34]. Whether there are other signaling pathways involved in neuroblastoma is a question worth exploring.

## Conclusion

Our results show that miR-429 inhibits neuroblastoma progression by downregulating the NF-κB signaling pathway. These are novel insights into how miR-429 serves as a tumor suppressor by targeting IKKβ and attenuating NF-κB activity. Furthermore, this may indicate that miR-429 is a new target for neuroblastoma therapy.

## Data Availability

Datasets used and/or analyzed in this study can be obtained from the corresponding author on reasonable request.

## Abbreviations

IKK: I kappa B kinase
Mut: Mutant
Wt: Wild-type

## References

[1] Maris JM, Hogarty MD, Bagatell R (2007). Neuroblastoma. Lancet.

[2] Oldridge DA, Wood AC, Weichertleahey N (2015). Genetic predisposition to neuroblastoma mediated by a LMO1 super-enhancer polymorphism. Nature.

[3] Pugh TJ, Morozova O, Attiyeh EF, Crimmins I, Sussman R, Winter C (2013). The genetic landscape of high-risk neuroblastoma. Nat Genet.

[4] Ward E, Desantis C, Robbins A, Kohler B, Jemal A (2014). Childhood and adolescent cancer statistics. Ca A Can J Clin.

[5] Westermann F, Schwab M (2002). Genetic parameters of neuroblastomas. Cancer Lett.

[6] Park JR, Bagatell R, London WB (2013). Children's oncology Group's 2013 blueprint for research: neuroblastoma. Pediatr Blood Cancer.

[7] Daniels JL, Olshan AF, Teschke K, Hertz-Picciotto I, Savitz DA, Blatt J (2001). Residential pesticide exposure and neuroblastoma. Epidemiology.

[8] Pacifico F, Leonardi A (2006). NF-kappaB in solid tumors. Biochem Pharmacol.

[9] Xiong S, Wang Y, Li H, Zhang X (2017). Low dose of Bisphenol a activates NF-κB/IL-6 signals to increase malignancy of neuroblastoma cells. Cell Mol Neurobiol.

[10] Karin M, Delhase M (2000). The I kappa B kinase (IKK) and NF-kappa B: key elements of proinflammatory signaling. Semin Immunol.

[11] Hayden MS, Ghosh S (2004). Signaling to NF-κB. Genes Dev.

[12] Häcker H, Karin M (2006). Regulation and Function of IKK and IKK-Related Kinases. Sci STKE.

[13] Kaisho T, Tanaka T (2008). Turning NF-κB and IRFs on and off in DC. Trends Immunol.

[14] Yu J, Wang L, Zhang T, Shen H, Dong W, Ni Y (2015). Co-expression of β-arrestin1 and NF-кB is associated with cancer progression and poor prognosis in lung adenocarcinoma. Tumour Biol.

[15] Karin M (2006). Nuclear factor-kappaB in cancer development and progression. Nature.

[16] Luo JL, Kamata H, Karin M (2005). IKK/NF-κB signaling: balancing life and death – a new approach to cancer therapy. J Clin Investig.

[17] Kasibhatla S, Brunner T, Genestier L, Echeverri F, Mahboubi A, Green DR (1998). DNA damaging agents induce expression of Fas ligand and subsequent apoptosis in T lymphocytes via the activation of NF-kappa B and AP-1. Mol Cell.

[18] Mayo MW, Baldwin AS (2000). The transcription factor NF-kappaB: control of oncogenesis and cancer therapy resistance. Biochim Biophys Acta.

[19] Meteoglu I, Erdogdu IH, Meydan N, Erkus M, Barutca S (2008). NF-KappaB expression correlates with apoptosis and angiogenesis in clear cell renal cell carcinoma tissues. J Exp Clin Cancer Res.

[20] Schmiedel JM, Klemm SL, Zheng Y, Sahay A (2015). MicroRNA control of protein expression noise. Science.

[21] Martinezsanchez A, Murphy CL (2013). miR-1247 functions by targeting cartilage transcription factor SOX9. J Biol Chem.

[22] Liu X, Peng H, Liao W, Luo A, Cai M, He J (2018). miR-181a/b Induces the Growth, Invasion and Metastasis of Neuroblastoma Cells Through Targeting ABI1. Mol Carcinog.

[23] Pereira DM, Rodrigues PM, Borralho PM (2013). Delivering the promise of miRNA cancer therapeutics. Drug Discov Today.

[24] Wu T, Lin Y, Xie Z (2018). MicroRNA-1247 inhibits cell proliferation by directly targeting ZNF346 in childhood neuroblastoma. Biol Res.

[25] Sun T, Wang C, Xing J, Wu D (2011). miR-429 modulates the expression of c-myc in human gastric carcinoma cells. Eur J Cancer.

[26] Ye ZB, Ma G, Zhao YH, Xiao Y, Zhan Y, Jing C (2015). miR-429 inhibits migration and invasion of breast cancer cells in vitro. Int J Oncol.

[27] Deng Y, Luan F, Zeng L, Zhang Y, Ma K (2017). MiR-429 suppresses the progression and metastasis of osteosarcoma by targeting ZEB1. EXCLI J.

[28] Liu D, Xia P, Diao D, Cheng Y, Zhang H, Yuan D (2012). MiRNA-429 suppresses the growth of gastric cancer cellsin vitro. J Biomed Res.

[29] Liu W, An J, Li K, Hou H (2015). MiR-429 regulates gastric cancer cell invasiveness through ZEB proteins. Tumour Biol J Int Soc Oncodevelopmental Biol Med.

[30] Zhang M, Dong BB, Lu M, Zheng MJ, Chen H, Ding JZ (2016). miR-429 functions as a tumor suppressor by targetingFSCN1in gastric cancer cells. Oncotargets & Therapy.

[31] Chen R, Alvero AB, Silasi DA, Kelly MG, Fest S, Visintin I (2008). Regulation of IKKβ by miR-199a affects NF-κB activity in ovarian cancer cells. Oncogene.

[32] Shin VY, Jin H, Ng EK, Cheng AS, Chong WW, Wong CY (2011). NF-κB targets miR-16 and miR-21 in gastric cancer: involvement of prostaglandin E receptors. Carcinogenesis.

[33] Arabzadeh S, Hossein G, Dulabi ZS, Zarnani AH. WNT5A–ROR2 is induced by inflammatory mediators and is involved in the migration of human ovarian cancer cell line SKOV-3. Cell Mol Biol Lett. 2016;21(1). 10.1186/s11658-016-0003-3 PMC5415827 28536612

[34] Kim S, Cheon H, Kim SM, Kim YY (2018). GSK-3β-mediated regulation of cadmium-induced cell death and survival. Cell Mol Biol Lett.

